# A Review of the Paradigmatic Role of Adipose Tissue in Renal Cancer: Fat Measurement and Tumor Behavior Features

**DOI:** 10.3390/cancers16091697

**Published:** 2024-04-27

**Authors:** Eliodoro Faiella, Elva Vergantino, Federica Vaccarino, Amalia Bruno, Gloria Perillo, Rosario Francesco Grasso, Bruno Beomonte Zobel, Domiziana Santucci

**Affiliations:** 1 Operative Reasearch Unit of Radiology and Interventional Radiology, Fondazione Policlinico Universitario Campus Bio-Medico di Roma, Via Alvaro del Portillo 200, 00128 Rome, Italy; e.faiella@policlinicocampus.it (E.F.); federica.vaccarino@unicampus.it (F.V.); amalia.bruno@unicampus.it (A.B.); gloria.perillo@unicampus.it (G.P.); r.grasso@policlinicocampus.it (R.F.G.); b.zobel@policlinicocampus.it (B.B.Z.); d.santucci@policlinicocampus.it (D.S.); 2 Research Unit of Radiology and Interventional Radiology, Department of Medicine and Surgery, Università Campus Bio-Medico di Roma, Via Alvaro del Portillo 21, 00128 Rome, Italy

**Keywords:** renal cancer, adipose tissue, fat measurements, VAT, PRAT, SAT, MAP score

## Abstract

**Simple Summary:**

This systematic review explores the relationship between obesity and renal-cell carcinoma (RCC), focusing on how different types of fat measurements influence the behavior and prognosis of the disease. Despite some studies suggesting a protective role of certain fat deposits, particularly visceral adipose tissue (VAT), conflicting findings exist across different adipose metrics and RCC subtypes. Methodological variations and limitations, such as retrospective designs and sample size constraints, pose challenges to standardization and generalizability. Further research efforts are needed to understand these associations better and establish standardized approaches for adiposity assessment in RCC patients, which could inform clinical practice and therapeutic decision-making.

**Abstract:**

(1) Background: Renal-cell carcinoma (RCC) incidence has been steadily rising, with obesity identified as a potential risk factor. However, the relationship between obesity and RCC prognosis remains unclear. This systematic review aims to investigate the impact of different adipose tissue measurements on RCC behavior and prognosis. (2) Methods: A search of MEDLINE databases identified 20 eligible studies focusing on various fat measurements, including visceral adipose tissue (VAT), subcutaneous adipose tissue (SAT), perirenal adipose tissue (PRAT), and the Mayo adhesive probability (MAP) score. (3) Results: The review revealed conflicting findings regarding the association between adipose tissue measurements and RCC outcomes. While some studies suggested a protective role of certain fat deposits, particularly VAT, against disease progression and mortality, others reported contradictory results across different adipose metrics and RCC subtypes. (4) Conclusions: Methodological variations and limitations, such as retrospective designs and sample size constraints, pose challenges to standardization and generalizability. Further research is needed to understand these associations better and establish standardized approaches for adiposity assessment in RCC patients, which could inform clinical practice and therapeutic decision-making.

## 1. Introduction

During the last two decades, there has been an annual increase of about 2% in renal-cell carcinoma (RCC) incidence both worldwide and in Europe, ranking sixth in men and tenth in women globally [1,2]. To date, several studies have investigated the association between obesity and RCC, notably with clear-cell histology. However, the impact on prognosis remains unclear.

Currently, optimal screening modalities and approaches have not yet been established [3]. Imaging modalities play a crucial role in the accurate detection of renal masses, with CT and MRI as referenced standards for renal lesion characterization [4,5,6].

While a higher body mass index (BMI) is recognized as risk factor for RCC [7], a paradoxical relationship exists, with some studies suggesting improved survival in obese RCC patients [8]. Obesity evaluation through imaging modalities is performed by CT scans, and measurements are obtained by the manual segmentation of adipose tissue in different anatomical sites. In fact, the intricate interplay between obesity and RCC prognosis is further complicated by the varying adiposity CT measures among studies, such as visceral adipose tissue (VAT), subcutaneous adipose tissue (SAT), perirenal adipose tissue (PRAT), and the Mayo adhesive probability (MAP) score. The “obesity paradox” observed in certain cancers prompts a nuanced exploration of RCC outcomes based on different adipose metrics [9]. This systematic review aims to gather all papers published to date concerning the relationship between perirenal fat and renal tumors, specifically exploring how different subtypes of fat measurements (VAT, SAT, PRAT, and MAP) influence stage, grade, histology, prognosis, and treatment success in renal cancer.

### 1.1. Obesity and Cancer

Obesity is a global health concern that is often associated with an increased risk for a wide range of malignancies [10]. The precise implications of obesity as a risk factor in cancer development and its influence on prognostic outcomes are not yet fully understood within the medical literature.The World Cancer Research Fund (WCRF) has identified ten cancers with established links to obesity. These include post-menopausal breast, endometrial, ovarian, advanced prostate [11], colorectal, renal, pancreatic, liver, gallbladder cancers, and esophageal adenocarcinoma [9]. Concerning prognosis, there is a prevailing notion that, in comparison to patients with a normal BMI, those with an elevated BMI may experience a poorer prognosis following all these types of cancer diagnosis. This observation is particularly evident in reviews of literature, especially among women with breast cancer [12].Nevertheless, some research have reported that among cancer patients, an elevated BMI is associated with improved survival compared to normal-weight counterparts. The unexpected nature of this discovery suggests the existence of an “obesity paradox” [9].

The intricate interplay between obesity and its potential contributions to the onset of cancer, as well as its subsequent effects on disease prognosis, requires further investigation and comprehensive analysis. 

### 1.2. Obesity and RCC

Body mass index (BMI) is widely recognized as a prominent risk factor for the development of renal-cell carcinoma (RCC), notably the clear-cell histological subtype [7]. Despite its association with an increased risk of RCC, there is evidence suggesting a protective role in prognosis [8]. The biological mechanisms underpinning the connection between obesity and kidney cancer, especially the potential protective role in disease progression or mortality, remain inadequately understood.

Moreover, there is concern about the methodology employed to evaluate excess adiposity [13]. While BMI is the most used measure, it is an imperfect indicator of adiposity, particularly in men due to their higher lean mass [14].

Increasingly employed tools include measurements such as the following:

- VAT (visceral adipose tissue) refers to the cross-sectional area of fat tissue measured, outlining the fat area in a single axial L3 slice, which strongly correlates with the overall amount of fat tissue in the body [15]. VAT is characterized by heightened cellularity, vascularity, and innervation and showcases an increased presence of inflammatory cells and large adipocytes [16].
- SAT (subcutaneous adipose tissue) refers to the subcutaneous fat area measured, outlining the fat area in a single scan obtained at the level of the umbilicus (approximately the level of L4 and L5) [17].
- PRAT (perirenal adipose tissue) refers to the perirenal adipose area obtained using a single axial slice at the level of the renal vein. The measurement involves the outlining the area of fat by manually tracing the limits of the anterior renal fascia laterally to the lateroconal ligament and including the posterior perirenal fat and retroperitoneal fat below Zuckerkandl’s fascia [18].
- PFT (perirenal fat thickness) is assessed by determining the perpendicular distance from the kidney’s posterior surface to the outer border of the iliopsoas muscle at the renal hilum level, employing the ruler tool within the relevant radiological software [19].
- MAP (Mayo adhesive probability) score, a validated and noninvasive tool for assessing perinephric fat “quality,” is currently associated with the presence of adherent perirenal fat encountered during partial nephrectomy (a non-tumor-related factor that can complicate PN) [20,21]. MAP score calculations are performed by measuring posterior perirenal thickness (the distance directly between the kidney capsule and the posterior abdominal wall at the level of the renal vein) and evaluating perinephric stranding. Posterior perirenal thickness lengths are assigned scores of 0, 1, or 2. Perinephric stranding at the renal vein level is assessed and assigned a score of 0 (none), 2 (type I), or 3 (type II) based on severity. The scores obtained for these two measurements are then totaled to derive the overall MAP score, ranging from 0 to 5 [22].

## 2. Materials and Methods

Two radiologists independently selected the studies and extracted data from each study using MEDLINE databases, such as PubMed and Web of Science, and the following strings: (“kidney cancer” OR “renal cancer” OR “renal cell carcinoma”) AND (“visceral adipose tissue” OR “subcutaneous adipose tissue” OR “perirenal adipose tissue” OR “MAP score”).

No limitations were applied to the search strategy.

Only studies which investigate the correlation between RCC and distinct types of fat measurements utilizing CT technique were included in the study.

Additionally, only publications in English, French, or Spanish with full-text availability and studies involving adult participants (aged 18 and older) were considered for inclusion.

Abstracts, reviews, case studies, letters to editors, comments, and editorials were excluded. Furthermore, studies involving pediatric populations were excluded. The last update of our database search was completed in August 2023.

For each study, the following information was collected: title, authors, publication year, journal, country, study design (retrospective or prospective), aim of the study, number of patients, age, gender, subtype of adipose measurement, histotype of tumor, tumor stage and grade, prognosis, and treatment.

**Figure 1:**
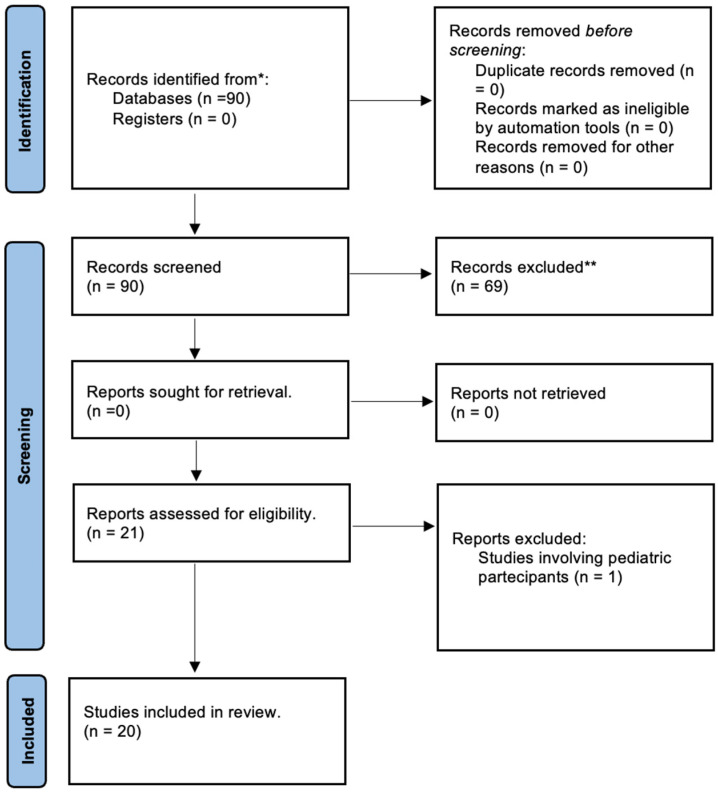
PRISMA 2020 flow diagram for the selection of studies included in the review. * Consider, if feasible to do so, reporting the number of records identified from each database or register searched (rather than the total number across all databases/registers). ** If automation tools were used, indicate how many records were excluded by a human and how many were excluded by automation tools.

In the process of composing the review, we adhered to the PRISMA statement guidelines; the checklist is available for reference in the Supplementary Materials, and the flow diagram for the selection of the studies included is summarized in Figure 1.

## 3. Results

From a total of 90 papers, 20 research articles were considered eligible; of these, two studies were prospective, and the others were all retrospective.

All these studies were published from 2010 to 2022. The study characteristics are shown in Supplementary Table S1. In the following paragraphs, we explore the role of different adipose tissue distribution patterns with RCC behavior.

### 3.1. VAT/SAT

In 2022, JSF Mauritis et al. [23] investigate the relationship between VAT, SAT, total adipose tissue (TAT), relative visceral adipose tissue (rVAT) and tumor stage and Fuhrman grade in RCC in a cohort of 1039 patients. Higher levels of visceral adipose tissue (VAT) and total adipose tissue (TAT) were significantly associated with a decreased risk of reaching stage III and stage IV, while elevated SAT correlated with a reduced risk of stage IV in both genders. Among males, increased rVAT was linked to a lower risk of stage IV. Females showed similar trends, but only VAT reached statistical significance. No significant associations were found for SAT. No linear associations between adipose tissue parameters and Fuhrman grade in both genders. However, females with SAT higher than the sex-specific median showed a lower risk of high Fuhrman grade.

Same results were obtained by other two studies. Y Naya et al. [24] observed that visceral VAT was notably higher in patients with stage I ccRCC compared to those with advanced stages. Additionally, individuals with elevated VAT exhibited significantly higher cumulative cause-specific survival rates in comparison to those with lower VAT levels. Furthermore, HW Lee et al. [25] revealed that higher VAT was associated with improved cancer-specific and overall survival in patients with advanced renal cell carcinomas undergoing nephrectomy. In advanced disease, low VAT independently predicted poor prognosis.

G Kaneko et al. [26] examined the prognostic impact of visceral obesity on 285 Japanese patients with localized ccRCC. High visceral fat area (≥120 cm^2^) correlated with better 5-year recurrence-free survival rates. Visceral fat area emerged as an independent predictor of recurrence-free survival, C-reactive protein, Fuhrman nuclear grade, tumor size and microvascular invasion. 

In their prospective study, JS Park et al. [27] investigated the link between visceral adiposity and target-gene mRNA expression in 200 patients with localized T1a stage clear-cell renal-cell carcinoma (ccRCC). The study found a significant association between high visceral adiposity and low ISUP grade. DDX11 mRNA expression in frozen tissue emerged as a potential biomarker, exhibiting a correlation with both visceral adiposity and high ISUP grade, suggesting its role in assessing aggressiveness in small ccRCC. 

Controversially, in 2020 Z Hu et al. [28] examine if sex-specific composition of abdominal visceral fat on CT scans can serve as a predictor for the Fuhrman nuclear grade in ccRCC on a cohort of 171 patients and revealed that rVAT emerged as an independent predictor for high-grade ccRCC with robust predictive power in females, while its predictive capability was not significant in males. 

Similarly, Y Zhu et al. [29], in their study on 186 patients with surgically treated cT1a renal cell carcinoma, identified a strong correlation between increased VAT% and higher Fuhrman grade.

A Keehn et al. [30] reported in a study involving 125 patients that increasing VAT and VAT% were significantly associated with worse preoperative MDRD (Modification of Diet in Renal Disease). In a subset multivariate analysis of 81 RCC patients, increasing VAT was statistically linked to worsening Fuhrman grade. However, no association was observed between SAT and tumor grade.

Furthermore, a study by GK Nguyen et al. [31] on 222 patients with ccRCC aimed to assess the impact of sex-specific abdominal visceral fat composition, measured by CT, and tumor glucose metabolism on patient outcomes. Women with a rVAT greater than 30.9% showed an increased risk of death, with glycolytic gene expression further stratifying survival outcomes for both genders. The combination of low rVFA and low glycolysis identified a subgroup of women with excellent overall survival.

Similarly, T Zhai et al. [32] investigated the association between VAT and short-term postoperative outcomes in ccRCC patients. The study revealed a positive correlation between higher VAT and increased surgical complexity, postoperative morbidity, prolonged postoperative stay, and higher hospitalization expenses.

YH Park et al. [33] examine the clinicopathological characteristics and assess the prognostic implications of VAT% in individuals diagnosed with localized RCC. Patients were stratified into quartiles (Q1 to Q4) according to relative VAT%. The study revealed a U-shaped association between VAT% quartiles and the risk of recurrence in RCC. The lowest VAT% quartile exhibited a higher prevalence of pT1 and low-grade disease, with an association between VAT% and high-grade disease but not high-stage disease. PFS varied significantly among VAT% quartiles, indicating an increased risk of recurrence in both the lowest and highest quartiles. The relationship between VAT% and RCC recurrence appears to follow a U-shaped pattern; gender-stratified analysis demonstrated a strong association between VAT% and RCC recurrence in males but not females.

R Mano et al. [34] investigated the connections between visceral and subcutaneous adiposity and clinicopathological features in non-metastatic clear cell renal cell carcinoma (ccRCC) patients within a Western population of 201 patients. In this study, no statistically significant associations were found between SAT or VAT and cancer stage and grade. 

HK Wang et al. [35] examined the association between visceral obesity and histological subtypes of renal cell carcinoma (RCC) in a multicenter Chinese cohort. Analysis of 487 patients revealed that ccRCC patients had a mean VAT 25 cm^2^ greater than non-ccRCC patients, emphasizing significant differences in VAT between the two subtypes. The study suggested that increased visceral fat could play a primary role in explaining the link between obesity and ccRCC, with VAT outweighing the effects of BMI and Type 2 diabetes in predicting RCC pathology.

### 3.2. MAP/PRAT

J Preza-Fernandes et al. [36] investigated the association between locally assessed fat depots and RCC progression and survival on 137 ccRCC patients. Perirenal adipose tissue (PRAT) area, influenced by age, VAT and BMI, showed upregulated UCP1 expression and smaller adypocytes compared with SAT. Larger PRAT areas and lower fat radiodensity were related with lower tumor grade; increased PRAT, both on the contralateral and tumor side, were associated with improved progression-free survival and overall survival, supporting the obesity paradox in RCC, where increased local fat depots appeared protective against disease progression and mortality.

Controversially in their study, comprising 174 localized clear cell renal cell carcinoma (ccRCC) patients, H Huang et al. [37] aimed to evaluate the association between perirenal fat thickness (PFT) and progression-free survival (PFS). Over a median follow-up of 38 months, high PFT was linked to worse PFS compared to low PFT (*p* = 0.005). In multivariable analysis, high PFT was independently associated with adverse PFS. 

In 2018 AP Bernstein et al. [22] examined the influence of perinephric fat on kidney cancer on 317 patients with renal masses. The qualitative measure, Mayo Adhesive Probability (MAP) score, but not PRAT, a quantitative measure, emerged as a predictor of malignant pathology. No association between MAP score or PRAT and Fuhrman grade. 

JP Tsai et al. [38] investigated the predictive value of different perinephric fat parameters for malignant pathology in renal tumors. Analyzing data from 109 patients, including perinephric fat volume (PFV), perinephric fat area (PFA), perinephric fat thickness (PFT), and Mayo adhesive probability (MAP) score, the study found that MAP score, PFV, PFA, and PFT were significantly increased in the malignant versus benign tumor group.

S Bier et al. [21] assessed the predictive value of the Mayo Adhesive Probability (MAP) score, renal pelvis score, and RENAL score in 280 patients undergoing laparoscopic partial nephrectomy. Both the RENAL score and MAP score demonstrated improved predictive capabilities for complications, while the renal pelvis score had a minor role. Notably, patients with a MAP score > 1 experienced significantly prolonged surgical time, and a cut-off value of ≥3 demonstrated 87.5% sensitivity for predicting peri-/postoperative complications. 

The prospective study by AJ Davidiuk et al. [20] aimed to develop an image-based nephrometry scoring system for predicting the presence of adherent perinephric fat (APF) during robot-assisted partial nephrectomy (RAPN). The study found that the MAP score accurately predicted the presence of APF, with increasing scores correlating with a higher likelihood of encountering APF during RAPN and longer operative times. 

The prospective study by DD Thiel et al. [39] aimed to evaluate the correlation between the MAP score and progression-free survival (PFS) in surgically treated patients with RCC. The MAP score was calculated for 456 patients. Patients with higher MAP scores (4–5) exhibited associations with male gender, older age, higher BMI, and larger tumors. Dichotomizing MAP scores into high (4–5) and low (0–3) revealed that elevated MAP scores (4–5) are linked to a reduction in PFS among surgically treated patients with clinically localized RCC, compared to patients with lower MAP scores (0–3).

## 4. Discussion

In 2016, the mortality rate for renal cell carcinoma (RCC) was approximately 2% of all cancer-related deaths [1,2]. Renal cancer presents various histological subtypes, each distinguished by a distinct molecular profile. In 2016, the WHO introduced a new classification for renal cell carcinomas (RCCs) based on the Vancouver consensus conference of the International Society of Urological Pathology (ISUP). This updated classification, based on cytoplasmic and architectural features, anatomic location, pathognomonic traits, and other characteristics, identifies 16 subtypes for RCC [40]. Three main RCC subtypes are clear cell (75% of cases [41]), papillary RCC (10–15%, type I and II), and chromophobe (4–5%). 

Notably, pRCC type I exhibits a lower nonmetastatic risk of death compared to ccRCC and pRCC type II [42]

BAP1 and PBRM1 gene expression, both on chromosome 3p, independently predict tumor recurrence, with BAP1-mutant tumors indicating worse outcomes. A 16-gene signature shows promise in predicting relapse [43,44].

Genome-wide studies reveal chromosomal alterations (e.g., gains in 7q, 8q, 20q, and losses in 9p, 9q, 14q) and CpG methylation-based assays providing independent survival predictions [45]. Prognostic insights from cytokines and immune checkpoint molecules like PD-L1 hold therapeutic potential yet require further exploration in RCC treatment [46]. Collectively, these factors contribute to enhanced diagnostic and prognostic precision in RCC.

Concerning histology subtypes, our review revealed that increased visceral fat might play a crucial role in explaining the connection between obesity and ccRCC: in particular high VAT (>25 cm^2^) is related with ccRCC rather than non-ccRCC histotype [35]. 

The Fuhrman grade is the most widely used grading system for certain types of kidney cancer including ccRCC and ppRCC [47]; it is based on microscopic features, including nuclear size, shape, and nucleoli prominence. Grades range from 1 to 4, with higher grades indicating more irregular nuclei and generally correlating with a worse prognosis [48]. 

Focusing on the Fuhrman grade, our study highlights the intricate relationship between adipose tissue parameters and RCC grading system.

While G Kaneko et al. [26] associated high visceral fat area with favorable outcomes and independent predictors of Fuhrman grade, in 2020 it was found that rVAT independently predicted high-grade clear cell renal cell carcinoma (ccRCC) with robust predictive power in females, while its predictive capability did not achieve significance in males [28]. 

In a more recent study (2022), in a large cohort of 1039 patients, JSF Mauritis et al. found no linear associations between adipose tissue parameters and Fuhrman grade, except for females with higher SAT displaying a lower risk of high Fuhrman grade [23].

Studies by Y Zhu et al. [29] and A Keehn et al. [30] reported conflicting correlations, associating increased visceral adipose tissue (VAT) and VAT% with higher Fuhrman grade and worse preoperative outcomes. It is essential to note that these are older articles from 2012 and 2014, respectively, and encompass relatively smaller patient cohorts, warranting consideration of these factors in the interpretation of the findings. 

In our review, a singular study investigated perinephric fat utilizing the MAP score, emphasizing its predictive significance in malignant pathology. Interestingly, no significant association with Fuhrman grade was observed in this context [38].

These findings underscore the complexity of the relationship between different adipose tissue measurements and RCC Fuhrman grading sysytem, necessitating further investigation and standardization for meaningful clinical implications.

In 2012, the WHO/ISUP introduced a four-grade system for renal cell carcinoma (ccRCC and ppRCC) [49,50], based on nucleolar prominence (grades 1–3) and the presence of highly atypical cells or specific morphologies (sarcomatoid or rhabdoid morphology) for grade 4, simplifying grading compared to Fuhrman’s system. These combined observations were incorporated into a novel grading classification for renal cell carcinoma to be known as ISUP Grading [51]. A recent prospective study (2021) previously mentioned in our review, analyzed VAT and mRNA expression as predictor of progression, considering ISUP grading system, in patients with localized T1a stage ccRCC. Patients were stratified into quartiles (Q1 to Q4) according to the relative VAT contents: the study revealed an association between the high quartile (indicative of high VAT) and low ISUP grade, along with decreased DDX1 expression in frozen tissue [27]. Although the study was prospective in nature, its limitation lay in the short duration of follow-up. Consequently, the researchers were unable to utilize visceral adiposity and target-gene expression to predict prognostic markers such as cancer-specific or progression-free survival in ccRCC patients. 

Among imaging modalities, US is the most common method for RCC detection, often diagnosed incidentally (about 50%) [52,53] with contrast-enhanced ultrasound (CEUS) evaluation considered as a precise and cost-effective tool for assessing indeterminate renal lesions. In cases suspected of malignancy, computed tomography (CT) scans or magnetic resonance imaging (MRI) are recommended [4]. Factors such as size, growth rate, fat content, contrast absorption pattern, and hyperattenuation in CT scans are key determinants of malignancy [5,6].

The 2017 TNM classification is the recommended standard for clinical and scientific staging [54,55]. TNM classification offers reliable anatomical information, complemented by nephrometry scores like R.E.N.A.L. (radius, exophytic/endophytic properties, nearness of tumor to the collecting system or sinus in millimeters, anterior/posterior, location relative to polar lines), aiming for standardized tumor assessment [56]. 

Concerning the relationship between fat measurements and RCC stage JSF Mauritis et al. [23] have also identified an inverse proportional relationship between VAT/TAT and stages III and IV, and between SAT and stage IV, with some differences between genders: among males, elevated rVAT was associated with a decreased risk of stage IV, females exhibited similar trends, although only VAT reached statistical significance, with no significant associations observed for SAT. Same results were obtained by Y Naya et al. [24] who observed an elevated risk of stage I in patients with high VAT%, in a cohort of 117 patients. 

Two conflicting studies were mentioned: one suggested a direct correlation between VAT and RCC stage, noting that the lowest quartile of VAT% was associated with a higher prevalence of pT1 and low-grade disease. However, in univariate logistic regression, they found an association between VAT% and high-grade disease but not with high-stage disease [33]. The limitation of this study, compared to the one conducted by Mauritius et al. [23], is that it focused on a cohort of patients with non-locally advanced or metastatic renal tumors, thus preventing the extension of these findings to all patient classes. The other (R Mano et al.) observed no association between SAT/VAT and tumor stage and grade, in a population of only 201 patients. Due to the small study sample, limited to the non-Western population, those results were not generalizable to Western populations. Additionally, due to the small number of deaths in this cohort, it was unable to perform a multivariable analysis to assess whether BMI and SFA were independent predictors of clinicopathological outcomes in RCC [34].

Anatomical, histological, clinical, and molecular factors provide crucial prognostic insights in renal cell carcinoma (RCC) [55]. 

In particular, among the aforementioned and described articles, a protective role of fat deposits (evaluated through various measurements) emerges in the majority of them, concerning progression, PFS, risk of recurrence, and mortality in renal tumors. 

More precisely, VAT is identified as an independent predictive factor of recurrence-free survival. Elevated VAT levels are associated with better 5-year RFS rates, higher cumulative cause-specific survival rates, and overall improved prognosis [26]. Two conflicting studies were mentioned: YH park et al. identified a U-shape pattern between VAT% quartiles and RCC risk of recurrence and PFS, but they did not investigate the impact of visceral obesity on cancer-specific mortality due to the inclusion of only patients with localized RCC, resulting in an insufficient number of deaths for analysis [33]; GK Nguyen et al. found an higher mortality risk in women with rVAT exceeding 30.9%. Since this study mainly looked at white patients, it’s hard to know how accurate the findings would be if applied to patients of other races who have lower rates of obesity [31]. 

Also, MAP score, PFV, PRAT and PFT are related with a higher risk of malignant versus benign tumor [38]. 

Specifically, the study by J Preza-Fernandes et al., supports the obesity paradox in RCC, indicating that larger PRAT areas may protect against disease progression and mortality, possibly through adipose-tumor cell interactions. Additionally, hypertensive patients have higher PRAT deposits on the tumor side, suggesting a common mechanism [36]. Contrarily, H. Huang et al. described a correlation between adverse PFS and elevated PFT [37]. However, their study’s focus on a small Chinese patient cohort (only 174 patients) limits its generalizability to other populations.

Therapeutic decisions should involve a multidisciplinary tumor board, including an interventional radiologist, urologist, and oncologist. 

The EAU guidelines suggest strong recommendations for renal tumor biopsy before therapy and percutaneous biopsy for active surveillance. Surgery is strongly advised for localized RCC, with partial nephrectomy for T1 tumors and embolization for unfit patients with significant symptoms. Laparoscopic radical nephrectomy is strongly recommended for T2 tumors, and lymph node removal during nephrectomy is endorsed.

Positive surgical margins and upstaged pT3a disease call for intensified follow-up. Thermal ablation or active surveillance is weakly recommended for frail patients with small masses [57]. According to CIRSE guidelines, T1a (≤4 cm) lesion ablations exhibit high technical success and excellent local control. Regarding primary treatment of T1b lesion, ablation exhibits a significantly higher risk of local recurrence compared to PN in this stage [58].

Post-treatment follow-up is recommended to monitor complications, renal function, local recurrence, and metastasis, with individualized, risk-based assessments based on prognostic factors.

Our review revealed different results regarding how fat deposits, in particular VAT and MAP score, influence post-treatment prognosis. 

Specifically, higher VAT was found to be associated with increased surgical complexity, postoperative morbidity, prolonged hospital stay, and higher healthcare costs. Since this study by T. Zhai et al. focused solely on Chinese and Asian populations, which generally have higher rates of visceral obesity compared to Western populations, these results cannot be extrapolated to Western populations [32]. 

Regarding the MAP score, a consistent finding across the aforementioned studies is that a higher MAP score is associated with an increased surgical risk in patients with renal tumors. In particular, MAP score greater than 1 had notably extended surgical duration, while a threshold of 3 or higher exhibited an 87.5% sensitivity in foreseeing peri-/postoperative complications, in patients undergoing laparoscopic partial nephrectomy [21]. Davidiuk et al. also observed a positive correlation between elevated MAP scores and increased surgical times during robot-assisted laparoscopic partial nephrectomy, highlighting the MAP score’s role in predicting the presence of adherent adipose tissue. This, along with the RENAL score, is suitable for imaging-based prediction of complex intraoperative conditions and short-term postoperative complications (assessed at 30 days), outperforming the RPS, particularly in predicting postoperative urinary leakage. Therefore, both the MAP score and the RENAL score serve as valuable tools for anticipating intraoperative and postoperative conditions [20]. Concerning the evaluation of the relationship between MAP score and PFS in patients undergoing surgery, higher MAP scores (4–5) are associated with decreased PFS in surgically managed patients with clinically localized RCC (pT1 RCC), in contrast to those with lower MAP scores (0–3) [39]. The limitation of this study lies in the lack of specification regarding the type of surgery undergone by patients, rendering it incomparable to the two studies mentioned above.

Limitations of these studies include:

- Limited number of articles and retrospective design: The scarcity of articles investigating correlations between RCC and fat deposits, combined with the predominance of retrospective designs, presents limitations in terms of sample size and potential biases in data collection.
- Variability and lack of standardization: There is a lack of standardized approaches across various facets, including diverse types of fat measurements, different RCC aspects, and variability in cohort populations. This variability impedes comparability and reproducibility of results. However, this wide variability also underscores the numerous aspects open to assessment and the potential for further advancement.

## 5. Conclusions

In conclusion, this systematic review underscores the complex relationship between obesity, adipose tissue distribution, and renal-cell carcinoma (RCC) behavior and prognosis. While some studies suggest a protective role of certain fat deposits, particularly visceral adipose tissue (VAT), against disease progression and mortality, conflicting findings exist across different adipose metrics and RCC subtypes. Methodological variations and limitations, such as retrospective designs and sample size constraints, pose challenges to standardization and generalizability. Further research efforts are warranted to elucidate the mechanisms underlying these associations and to establish standardized approaches for adiposity assessment in RCC patients, ultimately informing clinical practice and therapeutic decision-making.

## Data Availability

All data are available online.

## Supplementary Materials

The following supporting information can be downloaded at: https://www.mdpi.com/article/10.3390/cancers16091697/s1; Table S1: Characteristics of the studies included in the review. Title, authors, publication year, journal, country, study design (retrospective or prospective), aim of the study, number of patients, age, gender, subtype of adipose measurement, histotype of tumor, tumor stage and grade, prognosis, and treatment.
